# Marginally subcritical dynamics explain enhanced stimulus discriminability under attention

**DOI:** 10.3389/fnsys.2014.00151

**Published:** 2014-08-25

**Authors:** Nergis Tomen, David Rotermund, Udo Ernst

**Affiliations:** Institute for Theoretical Physics, University of BremenBremen, Germany

**Keywords:** criticality, neuronal avalanches, phase transition, attention, synchronization, gamma-oscillations, information entropy

## Abstract

Recent experimental and theoretical work has established the hypothesis that cortical neurons operate close to a critical state which describes a phase transition from chaotic to ordered dynamics. Critical dynamics are suggested to optimize several aspects of neuronal information processing. However, although critical dynamics have been demonstrated in recordings of spontaneously active cortical neurons, little is known about how these dynamics are affected by task-dependent changes in neuronal activity when the cortex is engaged in stimulus processing. Here we explore this question in the context of cortical information processing modulated by selective visual attention. In particular, we focus on recent findings that local field potentials (LFPs) in macaque area V4 demonstrate an increase in γ-band synchrony and a simultaneous enhancement of object representation with attention. We reproduce these results using a model of integrate-and-fire neurons where attention increases synchrony by enhancing the efficacy of recurrent interactions. In the phase space spanned by excitatory and inhibitory coupling strengths, we identify critical points and regions of enhanced discriminability. Furthermore, we quantify encoding capacity using information entropy. We find a rapid enhancement of stimulus discriminability with the emergence of synchrony in the network. Strikingly, only a narrow region in the phase space, at the transition from subcritical to supercritical dynamics, supports the experimentally observed discriminability increase. At the supercritical border of this transition region, information entropy decreases drastically as synchrony sets in. At the subcritical border, entropy is maximized under the assumption of a coarse observation scale. Our results suggest that cortical networks operate at such near-critical states, allowing minimal attentional modulations of network excitability to substantially augment stimulus representation in the LFPs.

## 1. Introduction

Self-organized criticality (SOC) is a property observed in many natural dynamical systems in which the states of the system are constantly drawn toward a critical point at which a phase transition occurs. A variety of systems such as sandpiles (Held et al., 1990), water droplets (Plourde et al., 1993), superconductors (Field et al., 1995), and earthquakes (Baiesi and Paczuski, 2004) exhibit SOC. In such systems, system elements are collectively engaged in cascades of activity called avalanches, whose size distributions obey a power-law at the critical state (Bak et al., 1987). Scientists have long hypothesized that SOC might also be a feature of biological systems (Bak and Sneppen, 1993) and that criticality of dynamics is relevant for performing complex computations (Crutchfield and Young, 1989; Langton, 1990). Support was given by modeling studies showing that networks of integrate-and-fire (IAF) neurons are able to display SOC (Corral et al., 1995), and predicting that avalanches of cortical neurons may belong to a universality class with a power-law exponent τ = 3/2 (Eurich et al., 2002).

Experimental data indicates that cortical dynamics may indeed assume a critical state: in 2003, Beggs and Plenz have shown that neuronal avalanche size distributions follow a power-law with τ = 3/2 in organotypic cultures as well as in acute slices of rat cortex. The observed avalanche size distributions hereby nicely matched the closed-form expressions derived for neural systems of finite size (Eurich et al., 2002). Subsequently, the ability of dissociated and cultured cortical rat neurons to self-organize into networks that exhibit avalanches *in vitro* was presented in Pasquale et al. (2008). Petermann et al. (2009) reported similar avalanche size distributions in the spontaneous cortical activity in awake monkeys. On a larger spatial scale, Shriki et al. (2013) presented scale-free avalanches in resting state MEG in humans. In addition, recent studies address questions relating to, for example, the rigorousness of statistical analysis (Klaus et al., 2011), subsampling (Priesemann et al., 2009), and resolution restraints as well as exponent relations (Friedman et al., 2012) in experimental criticality studies.

Combined, such theoretical and experimental results constitute the hypothesis that cortical neuronal networks operate near criticality (Bienenstock and Lehmann, 1998; Chialvo and Bak, 1999; Chialvo, 2004; Beggs, 2008; Fraiman et al., 2009). What makes the criticality hypothesis especially compelling is the idea that a functional relationship may exist between critical dynamics and optimality of information processing as well as information transmission (Bertschinger and Natschläger, 2004; Haldeman and Beggs, 2005; Kinouchi and Copelli, 2006; Nykter et al., 2008; Shew et al., 2009). However, the majority of neuronal avalanche observations are of spontaneous or ongoing activity in the absence of an actual sensory stimulus being processed by the cortex. In addition, no experimental studies exist to date which explore the criticality of neuronal dynamics *in vivo* in conjunction with a specific behavioral task, or under changing task demands.

Nevertheless, criticality describes the border between asynchronous and substantially synchronous dynamics, and in the field of vision research, synchronization has been studied extensively as a putative mechanism for information processing (von der Malsburg, 1994). Experimental studies demonstrated that in early visual areas, oscillations in the γ-range (about 40–100 Hz) occur during processing of a visual stimulus (Eckhorn et al., 1988; Gray and Singer, 1989). Hereby mutual synchronization between two neurons tends to become stronger if the stimulus components within their receptive fields are more likely to belong to one object (Kreiter and Singer, 1996), thus potentially supporting feature integration. Furthermore, it has been shown that selective visual attention is accompanied by a strong increase in synchrony in the γ-band in visual cortical networks (Fries et al., 2001; Taylor et al., 2005). In this context, γ-oscillations have been proposed to be the essential mechanism for information routing regulated by attention (Fries, 2005; Grothe et al., 2012). Moreover, recent studies have demonstrated links between synchronized activity in the form of oscillations in MEG (Poil et al., 2012) and LFP recordings (Gireesh and Plenz, 2008) and in the form of neuronal avalanches.

These findings motivated us to explore the potential links between synchronization, cortical information processing, and criticality of the underlying network states in the visual system. In particular, we investigated the criticality hypothesis in the context of γ-oscillations induced by selective visual attention. If visual cortical networks indeed assume a critical state in order to optimize information processing, such a state should be prominent during the processing of an attended stimulus, since attention is known to improve perception (Carrasco, 2011) and to enhance stimulus representations (Rotermund et al., 2009).

Specifically, we will focus here on a structurally simple network model for population activity in visual area V4. We will first demonstrate that our model reproduces key dynamical features of cortical activation patterns including the increase in γ-oscillations under attention observed in experiments (Fries et al., 2001; Taylor et al., 2005). In particular, we will explain how attention enhances the representation of visual stimuli, thus allowing to classify the brain state corresponding to a particular stimulus with higher accuracy (Rotermund et al., 2009), and we will identify mutual synchronization as the key mechanism underlying this effect.

Construction of this model allowed us to analyze dependencies between network states and stimulus processing in a parametric way. In particular, we were interested in whether such a network displayed critical dynamics, and how they relate to cognitive states. We inquired: Is criticality a “ground state” of the cortex which is assumed in the absence of stimuli, and helps process information in the most efficient way as soon as a stimulus is presented? Or is the cortex rather driven toward a critical state only when there is a demand for particularly enhanced processing, such as when a stimulus is attended?

For answering these questions, we (a) characterized the network state based on neuronal avalanche statistics (subcritical, critical, or supercritical), (b) quantified stimulus discriminability, and (c) analyzed the richness of the dynamics (information entropy of spike patterns) in the two-dimensional phase space spanned by excitatory and inhibitory coupling strengths. Within this coupling space, we identified a transition region where the network undergoes a phase transition from subcritical to supercritical dynamics for different stimuli. We found that the onset of γ-band synchrony within the transition region is accompanied by a dramatic increase in discriminability. At supercritical states epileptic activity emerged, thus indicating an unphysiological regime, and both information entropy and discriminability values exhibited a sharp decline.

Our main finding is that cortical networks operating at marginally subcritical states provide the best explanation for the experimental data (Fries et al., 2001; Taylor et al., 2005; Rotermund et al., 2009). At such states, fine modulations of network excitability are sufficient for significant increases in discriminability.

## 2. Results

### 2.1. Attention enhances synchronization and improves stimulus discriminability

Our study is motivated by an electrophysiological experiment (Rotermund et al., 2009) which has demonstrated that attention improves stimulus discriminability: While a rhesus monkey (*Macaca mulatta*) attended to one of two visual stimuli simultaneously presented in its left and right visual hemifields, epidural LFP signals were recorded in area V4 of the visual cortex. Power spectra of the Wavelet-transformed LFPs display a characteristic peak at γ-range frequencies between 35 and 80 Hz as well as a 1/*f* offset (**Figure 2A**). For assessing stimulus discriminability, Rotermund et al. used support vector machines (SVMs) on these spectral-power distributions in order to classify the stimuli on a single trial basis. A total of six different visual stimuli (complex shapes) were used in the experiments, therefore, the chance level was around 17%. This analysis yielded two results which are central for this paper:

1. Stimulus classification performance was significantly above chance level even in the absence of attention (35.5% for the V4 electrode with maximum classification performance).
2. Discrimination performance increased significantly (by 6.7% for the V4 electrode with maximum classification performance) when the monkey attended the stimulus inside the receptive field (RF) of the recorded neuronal population.

In this study, we present a minimal model which allows us to investigate putative neural mechanisms underlying the observed data.

### 2.2. Reproduction of experimental key findings

The spectra recorded in the experiment are consistent with neural dynamics comprising irregular spiking activity (the 1/*f*-background) and oscillatory, synchronized activity in the γ-band. In order to realize such dynamics in a structurally simple framework, we considered a recurrently coupled network of IAF neurons which is driven by Poisson spike trains. The network consists of both excitatory and inhibitory neurons interacting via a sparse, random coupling matrix with a uniform probability of a connection between two neurons (for details see Section 4.1). The strengths *J*_*inh*_ and *J*_*exc*_ of inhibitory and excitatory recurrent couplings are homogeneous. While oscillatory activity is generated as a consequence of the recurrent excitatory interactions, the stochastic external input and inhibitory couplings induce irregular spiking, thus providing a source for the observed background activity.

**Figure 1:**
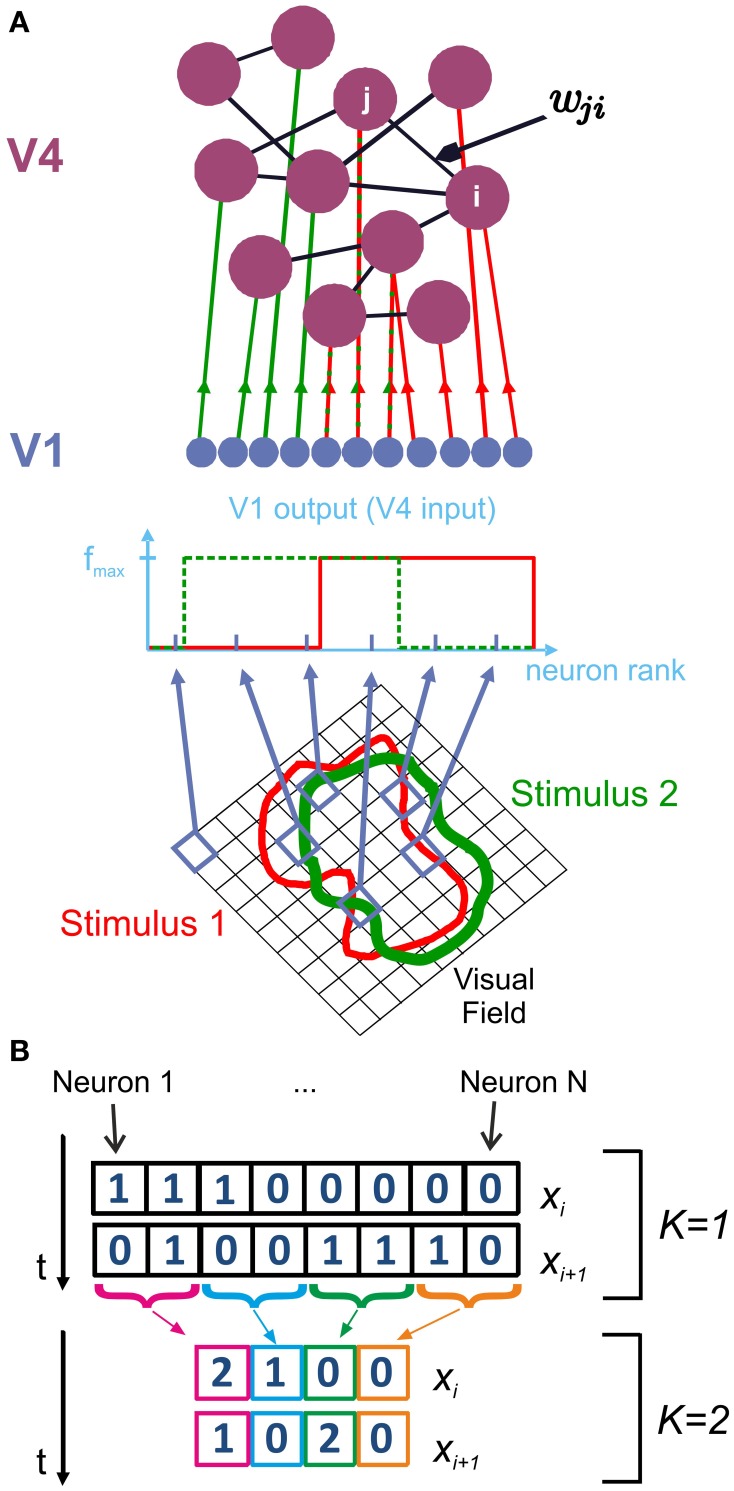
**Network structure and analysis of spike patterns**. We model V4 populations using a randomly coupled recurrent network of mixed excitatory (80%) and inhibitory (20%) integrate-and-fire neurons. **(A)** Depending on their receptive field properties, a different set of V1 neurons are activated by different stimuli. Activated V1 neurons provide feedforward input to V4 neurons *j* in the form of Poisson spike trains with rate *f*_*max*_. Consequently, a different, random subset of V4 neurons are driven by external input for each stimulus. Recurrent connections within V4 are represented by the random, non-symmetric coupling matrix *w*_*ji*_. **(B)** Information entropy of the spike patterns generated by area V4 is calculated using state variables *x*_*i*_. At the finest observation scale (*K* = 1), *x*_*i*_ consist of *N*-dimensional binary vectors, which represent whether each neuron *j* fired a spike (1) or not (0) at a given point in time. For larger *K*, the activity of *K* adjacent cells is summed to construct *x*_*i*_.

We consider this network as a simplified model of a neuronal population represented in LFP recordings of area V4 and the external Poisson input as originating from lower visual areas such as V1. One specific visual stimulus activates only a subset of V4 neurons by providing them with a strong external drive while the remaining V4 neurons receive no such input (Figure 1A). We drove a different, but equally sized subset of V4 neurons for each stimulus. Hence in a recording of summed population activity (e.g., LFPs), where the identity of activated neurons is lost, stimulus identity is represented in the particular connectivity structure of the activated V4 subnetwork. We simulated a total of *N* = 2500 neurons but kept the number of activated V4 neurons fixed at *N*_active_ = 1000 since every stimulus in the experiment was approximately the same size. With this setup, we ensured that the emerging stimulus-dependent differences in the network output are a consequence of stimulus identity and not of stimulus amplitude.

The variability of the couplings in our network mimics the structure of cortical couplings, which are believed to enhance certain elementary feature combinations [such as edge elements aligned to the populations' RF features (Kisvárday et al., 1997)] while suppressing others. Consequently, there will be stimuli activating subsets of V4 neurons which are strongly interconnected, while other stimuli will activate subsets which are more weakly connected.

We simulated the network's dynamics in response to *N*_*a*_ = 6 different stimuli in *N*_*tr*_ = 20 independent trials. Comparable to the experiments, LFP signals were generated by low-pass filtering the summed pre- and postsynaptic V4 activity (Section 4.1.3). We computed the spectral power distributions using the wavelet-transforms of LFP time series.

**Figure 2:**
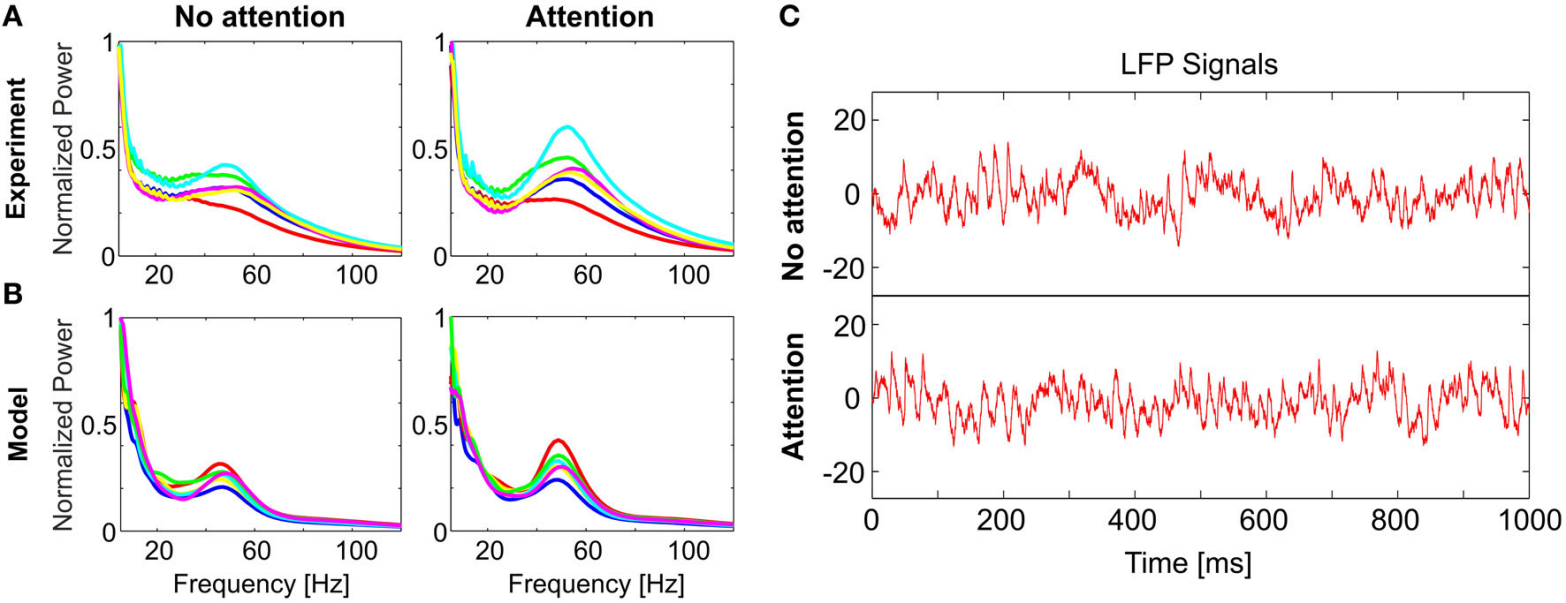
**Comparison of model dynamics to experimental recordings**. LFP spectral power distributions in **(A)** the experiment and **(B)** the model for non-attended (left) and attended (right) conditions. In each case, spectra averaged over trials is shown for 6 stimuli (different colors). In both **(A,B)** the spectra for each stimulus is normalized to its respective maximum in the non-attended case. Model spectra reproduce the stereotypical 1/*f* background as well as the γ-peaks observed in the experimental spectra. Under attention, γ-band oscillations become more prominent and spectra for different stimuli become visibly more discriminable. **(C)** Single trial LFP time-series from the model, illustrating the analyzed signals in the non-attended (top) and attended (bottom) conditions. [Data shown in **(A)** is courtesy of Dr. Andreas Kreiter and Dr. Sunita Mandon and Katja Taylor (Taylor et al., 2005)].

For sufficiently large *J*_*exc*_ the neurons in the V4 population were mutually synchronized, leading to a peak in the power spectra at γ-band frequencies. The average frequency of the emergent oscillations depends mainly on the membrane time constant τ for the particular choice of external input strength. Averaged over trials, these power spectra reproduced all the principal features displayed by the experimental data (Figure 2). In particular, spectra for individual stimuli differed visibly, with largest variability observed in the γ-range. Since the identity of activated neurons is lost in the population average, any differences in strength of the observed γ-oscillations can only be attributed to subnetwork connectivity. This result has a natural explanation because connection strength and topology strongly determine synchronization properties in networks of coupled oscillatory units (see for example Guardiola et al., 2000; Lago-Fernández et al., 2000; Nishikawa et al., 2003).

Differences in power spectra become even more pronounced if a stimulus is attended. We modeled attention by globally enhancing excitability in the V4 population. This can be realized either by increasing the efficacy of excitatory interactions, or by decreasing efficacy of inhibition. In this way, the gain of the V4 neurons is increased (Reynolds et al., 2000; Fries et al., 2001; Treue, 2001; Buffalo et al., 2010), and synchronization in the γ-range gets stronger and more diverse for different stimuli while the 1/*f*-background remains largely unaffected (Figure 2B). For visualizing the effect of attention, single trial LFP signals corresponding to attended and non-attended conditions for a specific stimulus are given in Figure 2C. Note that the change induced by attention does not need to be large; in the example in Figure 2 inhibitory efficacy was reduced by 10% from *j*_*inh*_ = 0.80 to 0.72.

The observed changes in the power spectra with attention can be interpreted in terms of the underlying recurrent network dynamics: each activated subnetwork has a particular composition of oscillatory modes, and enhancing excitability in such a non-linear system will activate a larger subset of these modes more strongly. This effect is enhanced by synchronization emerging at different coupling strengths for different stimuli. With a further increase in the coupling, however, groups of neurons oscillating at different frequencies will become synchronized at a single frequency (Arnold tongues, Coombes and Bressloff, 1999), which ultimately decreases the diversity of power spectra.

### 2.3. Enhancement of stimulus discriminability is a robust phenomenon

**Figure 3:**
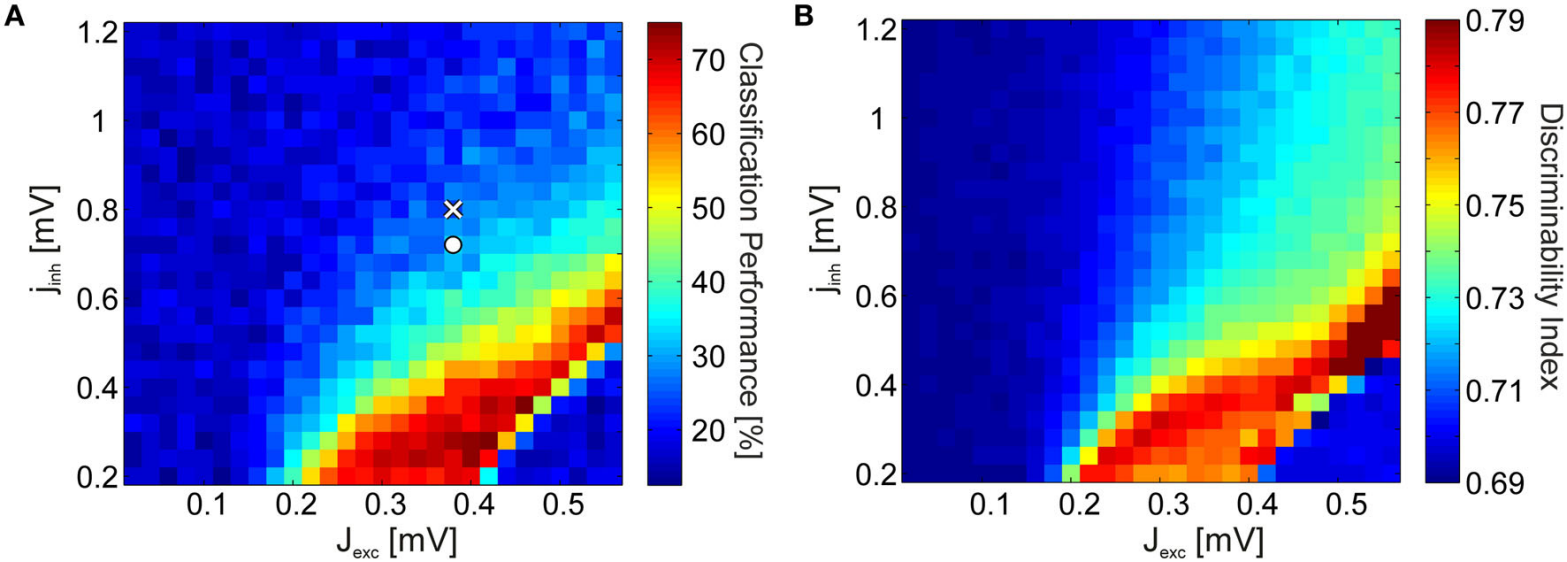
**SVM test results and the discriminability index. (A)** SVM classification performance as a function of the excitatory coupling strength *J*_*exc*_ and the inhibitory coupling scaling factor *j*_*inh*_ (obeying *J*_*inh*_ = ϵ · *J*_*exc*_ · *j*_*inh*_). The coupling values representing the non-attended and attended conditions in Figure 2B are marked by a cross and a circle, respectively. **(B)** Discriminability index in the coupling space for the same spectra. For both **(A,B)**, the strength of the background noise was *c*_*mix*_ = 0.2.

The spectra in Figure 2B were generated using coupling parameters *J*_*exc*_ and *J*_*inh*_ specifically tuned for reproducing the experimental data. However, the basic phenomenon is robust against large changes in the parameters: Discriminability increase is coupled to the emergence of strong γ-oscillations. To show this, we varied the excitatory and inhibitory coupling strengths independently, and quantified stimulus discriminability using SVM classification for every parameter combination. When varying the inhibitory efficacies, we used a step size that is proportional to the excitatory efficacy: *J*_*inh*_ = ϵ · *J*_*exc*_ · *j*_*inh*_ for every point in the coupling space where *j*_*inh*_ is the inhibitory scaling factor. We set the upper bound of excitation and the lower bound of inhibition so as to avoid unphysiologically high firing rates due to the activation of all neurons, including those that did not receive external input. Figure 3A shows the classification results in coupling space, averaged over *N*_*w*_ = 5 independently realized random connectivity architectures of the V4 network. The coupling values used for generating the spectra in Figure 2B are indicated by white markers. Classification performance is 24.2% in the non-attended (white cross) condition (significantly above chance level, ~17%, via a one-tailed binomial test with *p* < 0.005) and 32.8% in the attended (white circle) condition. Notably, discriminability is significantly above chance level only in a bounded region of the parameter space. Within this region, relatively small increases in excitatory, or decreases in inhibitory coupling strengths lead to an acute discriminability enhancement.

This effect comes about in the following way: In networks with low excitation and high inhibition, the dynamics are asynchronous and the LFP spectra are dominated by the 1/*f*-noise. In this case, every stimulus input is mapped to a network output with similar spectral components and with a large trial-to-trial variance. This severely impedes the ability to classify stimuli correctly. On the other hand, in networks with very high excitation and low inhibition, synchronous activity dominates the dynamics and epileptic behavior is observed. Mutual synchronization of the activated V4 neurons leads to co-activation of the otherwise silent V4 neurons which do not receive external input. This means that every stimulus input is mapped to spike patterns where almost all neurons are simultaneously active at all times. The corresponding spectra have reduced trial-to-trial variability but are almost identical for different stimuli. Consequently, stimulus discriminability reaches a maximum only in a narrow region of the parameter space which is associated with the onset of synchrony.

It is necessary to point out that the absolute magnitude of the SVM performance depends strongly on the background noise (i.e., on the value of *c*_*mix*_) which constitutes the 1/*f*-background in the spectra. For example, without the addition of the background noise (i.e., *c*_*mix*_ = 0), SVM classification performance is 36.67% for the non-attended and 43.83% for the attended spectra in Figure 2B. Nevertheless, the observation of a bounded region of enhanced discriminability persists even in the absence of 1/*f*-noise. This finding has an important consequence: It allows us to identify coupling parameters which cannot explain the experimental data regardless of the “real” noise level. Thus, it outlines a specific working regime in which the model can reproduce both of the experimental findings described in Section 2.1.

### 2.4. Characterization of dynamical network states

Our findings indicate that a significant discriminability increase correlates implicitly with the onset of synchronous dynamics. In the following, we will focus on this network effect in more detail, and investigate its ramifications for information processing in the visual system.

In order to obtain a better understanding of the behavior of the system, we implemented certain reductions to our simulations. First, we excluded regions in parameter space where all neurons not receiving external input became activated. For most of the phase space, recurrent excitation is not strong enough to activate these stimulus-nonspecific neurons. At the supercritical regions, where excitation is strong and neurons are firing synchronously, however, these silent neurons become activated. This effect further increases the average excitatory input strength in the recurrent V4 population, leading to epileptic activity at very high (biologically implausible) frequencies. Such a regime would be highly unrealistic, since neurons in V4 populations have well-structured receptive fields and are only activated by specific stimuli (Desimone and Schein, 1987; David et al., 2006). Therefore, we proceeded to isolate the activity of externally driven subnetworks and focused our analysis on their output. This was realized by limiting the number of neurons in the network to *N* = *N*_active_ = 1000 and by assigning different random coupling matrices to simulate different stimulus presentations. Thus, distinct network architectures stand for distinct stimulus identities.

When constructing the output signal, we now excluded the background noise induced by the V1 afferents (i.e., we set *c*_*mix*_ = 0), but note that the V4 neurons were still driven by this stochastic, Poisson input. This segregation of V4 activity from background noise was necessary for the analysis of network dynamics, in order to ensure that the observed variance of the LFP spectra across trials originated in the V4 population.

In the reduced simulations, spikes propagated and impacted the postsynaptic neurons' membrane potentials instantaneously (see Section 4.3). We also prevented neurons from firing twice during an avalanche. These latter changes were introduced for inspecting criticality in the system dynamics (described in detail in Section 2.4.1), allowing us to quantify the number of neurons involved in an avalanche event accurately.

Since SVM classification is a comparatively indirect method for quantifying discriminability, employing classifiers which are difficult to interpret, we introduce the discriminability index (DI) as a simplified measure. The DI quantifies by how much, averaged over frequencies, the distributions of LFP spectra over trials overlap for each stimulus pair (see Section 4.2.3). As oscillations emerge in network dynamics, trial-to-trial variability of the spectra decrease (i.e., width of the distributions become narrower), and the average spectra for each stimulus is more distinct (i.e., the means of the distributions disperse). Hence, DI provides us with a meaningful approximation of the SVM classification performance. We find that the DI yields a phase space portrait (Figure 3B) similar to the SVM classification result (Figure 3A) for the full network simulations.

**Figure 4:**
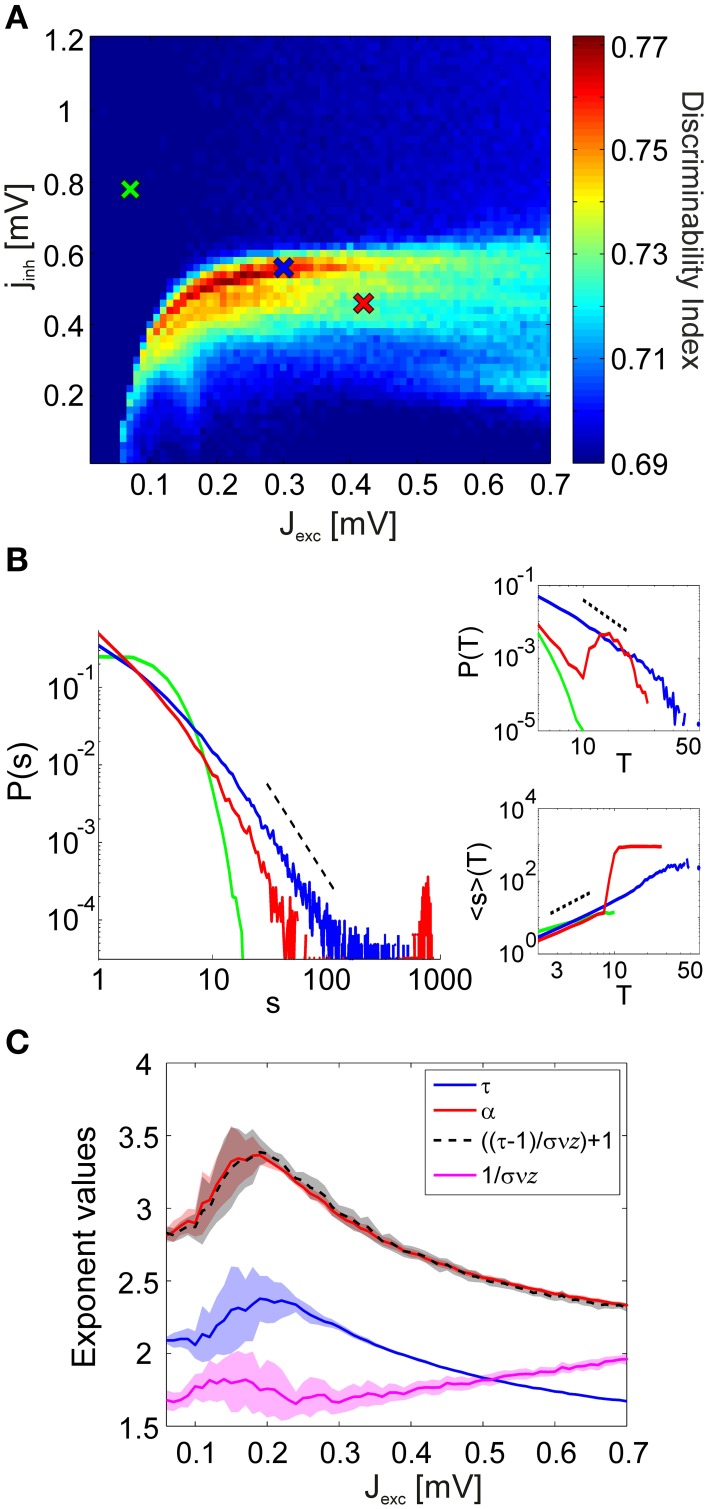
**Discriminability of the LFP spectra in relation to the avalanche statistics. (A)** Discriminability index in the reduced simulations. As in the full simulations (Figure 3B), stimulus discriminability increases dramatically in a narrow region of the coupling space. **(B)** Avalanche size distributions *P*(*s*) in the sub-critical (green), critical (blue), and super-critical (red) regimes for a single stimulus. Insets show how the corresponding avalanche duration distributions *P*(*T*) and the mean avalanche sizes 〈*s*〉 conditioned on the avalanche duration *T* behave in the three distinct regimes. The corresponding coupling parameter values are marked with crosses in **(A). (C)** The values of the estimated power-law exponents τ, α, and 1/σν*z* for each value of the excitatory coupling strength *J*_*exc*_. The lines mark the mean exponent at the critical point for each stimulus and the corresponding colored patches represent the standard deviation over the stimuli. The black dashed line shows the value of α computed using Equation 3, by plugging in the other two exponents.

In order to compute discriminability in the reduced simulations, we used *N*_*tr*_ = 36 trials from each of the *N*_*a*_ = 20 different stimuli. Simulations with the reduced network produce the same qualitative behavior in phase space (Figure 4A), in the sense that discriminability increase is only observed in a narrow region in the phase space, located in the border between regimes with and without strongly synchronous activity. Discriminability is maximized as oscillations emerge, and decays quickly in the regions where epileptic behavior is observed as all neurons fire simultaneously. Combined with the experimental evidence, our findings suggest that the cortex operates near a particular state where small modifications of excitability lead to substantial changes in its collective dynamics.

However, time-averaged power spectra of local field potentials are not well suited for characterizing different aspects of this state. Since epidural LFPs are signals averaged over large neuronal populations, dynamic features in spiking patterns become obscured, and temporal variations in the network dynamics are lost in the averaging process. In the following, we will go beyond LFPs and focus on (a) the size distribution of synchronized events (avalanche statistics), and (b) on the diversity and richness of patterns generated by the network (measured by information entropy).

#### 2.4.1. Criticality of dynamics

The network dynamics can be classified into three distinct regimes of activity characterized by their avalanche size distributions: subcritical, critical, and supercritical (Figure 4B). In the subcritical state spiking activity is uncorrelated, events of large sizes are not present and the probability distributions *P*(*s*) of observing an avalanche event of size *s* exhibit an exponential decay. In the supercritical state, spiking activity is strongly synchronous and avalanches spanning the whole system are observed frequently. This behavior is represented in the avalanche size distributions by a characteristic bump at large event sizes. The critical state signifies a phase transition from asynchronous to oscillatory activity and the corresponding avalanche size distributions *P*(*s*) display scale-free behavior.

(1) $$P(s)\propto s^{-\tau }$$

Even though power-law scaling of the avalanche size distributions, combined with the sudden emergence of oscillatory behavior in the system strongly suggest a phase transition in network dynamics, it is not sufficient to definitively conclude that the system is critical (Beggs and Timme, 2012; Friedman et al., 2012). Therefore, for inspecting criticality in the network dynamics, we have investigated the behavior of two other, relevant avalanche statistics: the distribution *P*(*T*) of avalanche durations *T* and the mean avalanche size 〈*s*〉 given the avalanche duration *T*, 〈*s*〉(*T*). We find that both of these distributions follow a power-law for intermediate values of *T* at the critical points (Figure 4B, insets).

(2) $$P(T)\;\propto T^{-\alpha }$$

(3) $$\langle s\rangle (T)\;\propto T^{1/\sigma \nu z}$$

We observe that the behavior of *P*(*T*) within the phase space is similar to that of *P*(*s*). In the subcritical regime, there are only avalanches of short durations, and *P*(*T*) has a short tail. In the supercritical regime, *P*(*T*) displays a bump at large event durations. For 〈*s*〉(*T*), we observe scale-free behavior of the distributions in both subcritical and critical regimes. Again a bump appears for large *T* at the supercritical regimes. In order to quantify the power-law scaling of the avalanche size and duration distributions we applied a maximum-likelihood (ML) fitting procedure (Clauset et al., 2009) and obtained an ML estimation of the power-law exponent for every stimulus. We obtained the power-law exponent of the mean size distributions conditioned on the avalanche duration using a least squares fitting procedure (Weisstein, 2002). Notably, the exponents obtained from the simulated dynamics fulfill the exponent scaling relationship (Figure 4C)

(4) $$\frac{\alpha -1}{\tau -1}=\frac{1}{\sigma \nu z}$$

as predicted by universal scaling theory (Sethna et al., 2001; Friedman et al., 2012).

**Figure 5:**
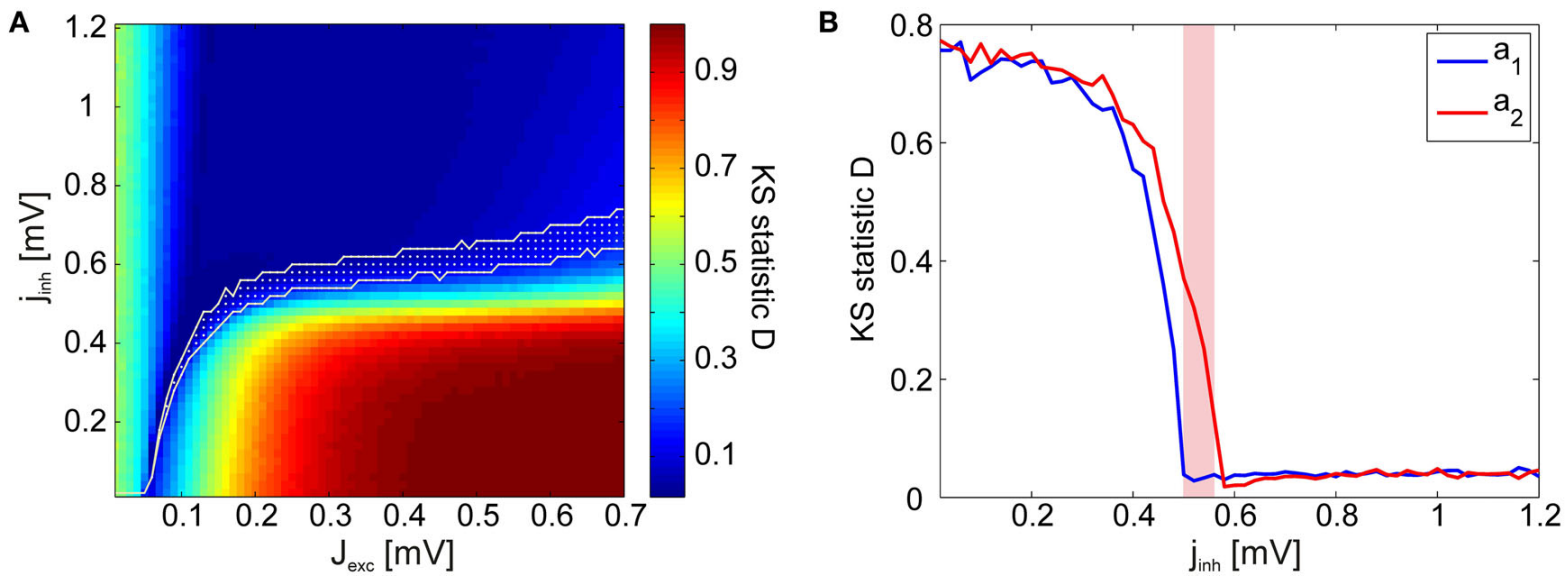
**KS statistic as a measure of criticality. (A)** KS statistic *D* of the avalanche size distributions in the reduced network, averaged over all stimulus presentations. Visual inspections reveal that the avalanche size distributions *P*(*s*) are characteristically subcritical (exponential) for most points in the coupling space with low *D*-values. The transition region calculated using the γ measure is given in white. **(B)** KS statistic *D* as a function of inhibitory coupling scaling factor *j*_*inh*_ for two exemplary stimuli, *a*_1_ (blue) and *a*_2_ (red), illustrating how the *D* minima occur at different points in the phase space for different stimuli (*J*_*exc*_ = 0.2 mV). The γ-transition region is given in magenta.

As a goodness-of-fit measure for the avalanche size distributions, we employed the Kolmogorov–Smirnov (KS) statistic. The KS statistic *D* averaged over all stimuli (i.e., network architectures) is given in Figure 5A. However, for identifying points in the phase space at which the network dynamics are critical, the KS statistic is ineffective: Even in the transition region from subcritical to supercritical behavior, the avalanche size distributions rarely display a perfect power-law which extends from the smallest to the largest possible event size. Therefore, we introduced lower and upper cut-off thresholds on the avalanche sizes during the fitting process (see Section 4.3). While this procedure allowed us to do better fits, it also lead to a large region of subcritical states which had relatively low (and noisy) *D*-values. This presents a predicament for automatically and reliably detecting the critical points by searching for minima in the *D*-landscape. Furthermore, we found that avalanche size distributions become scale-free at different points in phase space for different stimuli (Figure 5B). Therefore, the minima of the average KS statistic in Figure 5A are not representative of the critical points of the system.

**Figure 6:**
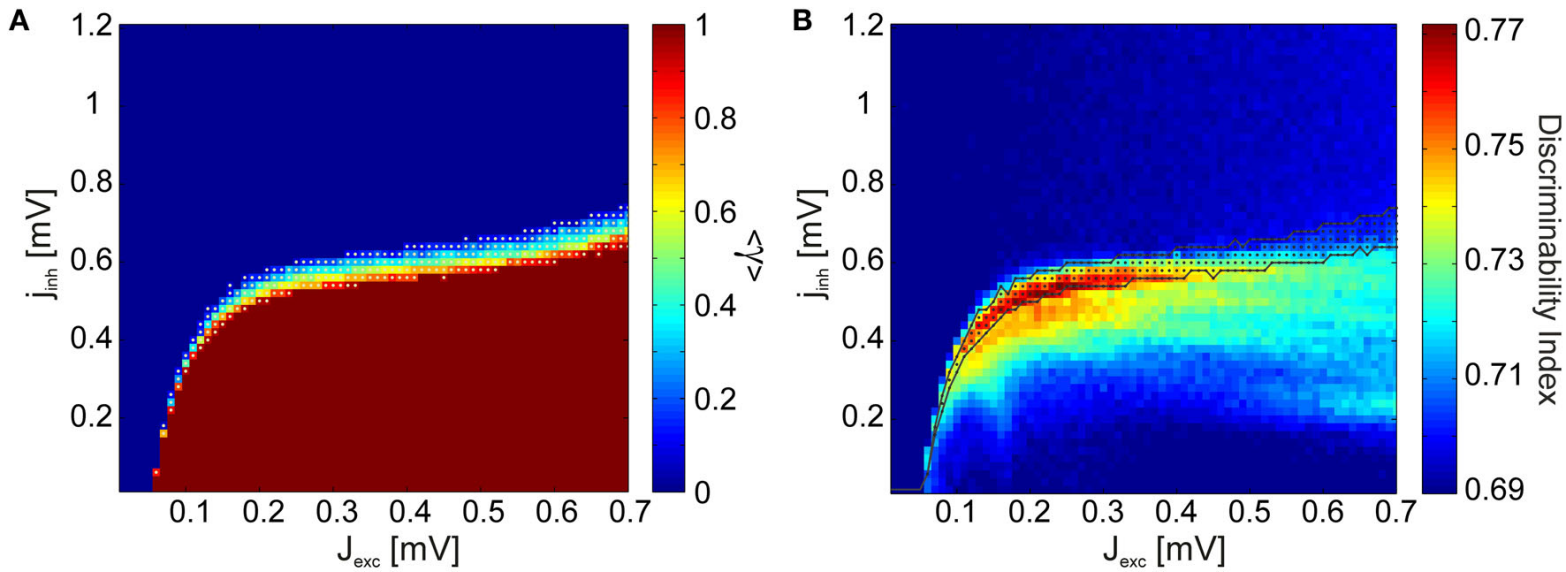
**Discriminability is enhanced in the region defining a phase transition from subcritical to supercritical avalanche statistics. (A)** γ measure averaged over all stimulus presentations. The network dynamics are subcritical for all stimuli in the regions of the phase space where the mean 〈γ〉 = 0, and supercritical in the regions where 〈γ〉 = 1. A phase transition from subcritical to supercritical dynamics takes place between these two regions, at different points for different stimuli. This transition region where 0 < 〈γ〉 < 1 is indicated by white dots. **(B)** Comparison of the discriminability index (Figure 4A) and the transition region.

Visual inspections revealed that the subcritical avalanche size distributions converge slowly to a power-law as inhibition is decreased. At a critical value of inhibition, a phase transition occurs and the bump characteristic of supercritical distributions appears abruptly. Consequently, it is trivial to determine the transition regions graphically. We automatized this procedure by using a binary variable γ, which assumes a value of 1 if a bump is detected in the avalanche size distributions (if the distribution is supercritical) and 0 otherwise (if the distribution is subcritical). Its mean 〈γ〉 over all stimuli is given in Figure 6A. We observed that there are clearly defined regions of sub- and supercritical dynamics, where γ is 0 or 1 for all stimuli, respectively. The points for which 0 < 〈γ〉 < 1 define the transition region, where synchronization builds up rapidly for different stimuli.

In Figure 6B the transition region is plotted together with the discriminability index for comparison. We observe that the points at which discriminability is enhanced are confined to the neighborhood of the transition region. Discriminability is maximized within the transition region, where the network dynamics are supercritical for a subset of architectures and subcritical for the remaining ones. This means that if cortical neurons were to maximize discriminability, a set of stimulus inputs would effectively map to epileptic output activity. Such a scenario is not only physiologically implausible, but actually pathological. Taken together, these findings suggest that only marginally subcritical points, and not ones within the transition and supercritical regions, qualify for explaining the experimental observations.

Therefore we propose that the cortex operates at near-critical states, at the subcritical border of the transition region. Such near-critical states are unique in their ability to display significant discriminability enhancement under attention while avoiding pathologically oscillatory dynamics. In addition, strongly correlated activity is associated with encoding limitations. However, neither the discriminability of LFP spectra, nor the avalanche statistics considered putative, neurophysiologically plausible decoding schemes used by downstream visual areas. To address this issue, we next inspected the diversity of spike patterns generated in the V4 network, and how this diversity behaves in the neighborhood of the transition region.

#### 2.4.2. Information entropy

We computed information entropy (Shannon, 1948) in order to assess the diversity of V4 spike patterns generated in response to stimuli within the coupling space. In doing so, we considered different scales on which read-out of these patterns, e.g., by neurons in visual areas downstream of V4, might take place.

At the finest scale of observation, the read-out mechanism has access to complete information about V4 spiking activity. In this case, it can discriminate between spikes originating from distinct presynaptic V4 neurons. At the coarsest observation scale, the read-out mechanism is not capable of observing every individual neuron, but rather integrates the total V4 input by summing over the presynaptic activity at a given time. To account for this, we introduce a scale parameter *K* which reduces a spike pattern **X** comprising spikes from *N* neurons to a representation of *N*/*K* channels with each channel containing the summed activity of *K* neurons (Figure 1B).

**Figure 7:**
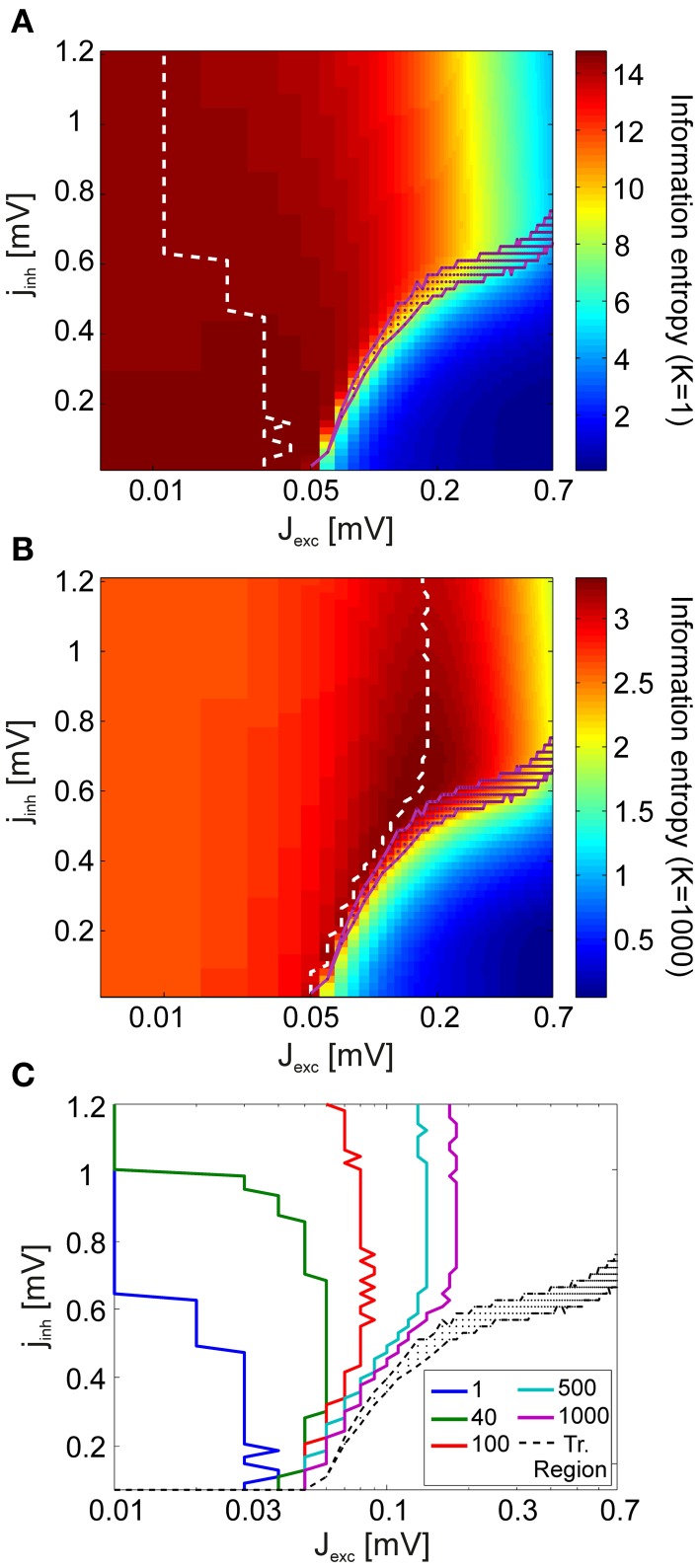
**Analysis of information entropy in V4 spike patterns**. Information entropy in coupling space for the finest observation scale (*K* = 1) **(A)** and the coarsest observation scale (*K* = *N* = 1000) **(B)** averaged over all stimuli. In **(A,B)**, the dashed white lines indicate the entropy maxima for each value of the inhibitory coupling scaling factor *j*_*inh*_. The magenta dots mark the transition region. **(C)** The maxima of information entropy for different observation scales *K*. Entropy maxima converge toward the transition region (black) as *K* is increased.

Figures 7A,B show how information entropy compares with the transition region of the system for *K* = 1 (full representation) and for *K* = *N* (summed activity over whole network). For each inhibitory coupling, the value of the excitatory coupling which maximizes information entropy is marked with a dashed line. For both conditions, we see that information entropy displays a sharp decline near the transition region. This behavior is consistent with a phase transition toward a regime of synchronous activity as the emergence of strong correlations attenuate entropy by severely limiting the maximum number of possible states. In comparison to the finest scale of observation (*K* = 1), we find that the maxima of information entropy are shifted to greater values of excitation at the coarsest scale of observation (*K* = *N* = 1000). Figure 7C shows how the maxima of information entropy evolve as a function of observation scale *K*, converging onto near-critical points. This effect arises because, as *K* increases, the points with the greatest number of states in the network activity are shifted toward the transition region. By construction, the number of possible states of *X* is finite, and the uniform distribution has the maximum entropy among all the discrete distributions supported on the finite set {*x*_1_, …, *x*_*n*_}. Hence, information entropy of the spike patterns increases with both an increase in the number of observed states and an increase in the flatness of the probability mass function *P*(**X**) of the states. For the coarsest scale of observation, *P*(**X**) is equivalent to the avalanche size distributions, and it is clear that a power-law scaling of these distributions cover the largest range of states (Figure 4B). However, for large *j*_*inh*_ (*j*_*inh*_ ≳ 0.6), entropy maxima persist at moderately subcritical regions. For large *K*, these regions are characterized by *P*(**X**) with smaller supports but more uniform shapes than the *P*(**X**) near the transition region. The flatness of these distributions, especially at small event sizes, causes the entropy maxima to appear around *J*_*exc*_ = 1.8 mV, instead of being located at higher values of excitation.

Combined, our results can be interpreted in the following way for the two extreme conditions discussed:

1. If neurons in higher areas of the visual system perform a spatial integration of the neuronal activity in the lower areas (*K* large), V4 networks operating at near-critical regimes both maximize information entropy and achieve significant discriminability enhancement under attention.
2. If V4 neurons employ a more efficient encoding strategy, where both spike times and neuron identities contain meaningful information for higher areas (*K* small), entropy is maximized by subcritical states with asynchronous dynamics. In such a scenario near-critical states represent a best-of-both-worlds optimization. At the subcritical border of the transition region, onset of oscillations and discriminability enhancement can manifest while avoiding a drastic loss in information entropy.

## 3. Discussion

In this paper we addressed the criticality hypothesis in the context of task-dependent modulations of neuronal stimulus processing. We focused, in particular, on changes in cortical activity induced by selective visual attention. We considered recent findings that γ-band oscillations emerge collectively with an enhancement of object representation in LFPs in macaque area V4 under attention (Rotermund et al., 2009). We reproduced these results using a model of a visual area V4 population comprising IAF neurons recurrently coupled in a random network. Attention induces synchronous activity in V4 by modulating the efficacy of recurrent interactions. In the model, we investigated the link between experimentally observed enhancement of stimulus discriminability, scale-free behavior of neuronal avalanches and encoding properties of the network quantified by information entropy.

We found that the emergence of γ-band synchrony is strongly coupled to a rapid discriminability enhancement in the phase space. Notably, we observed that discriminability levels comparable to the experiments appear exclusively in the neighborhood of the transition region, where network dynamics transition from subcritical to supercritical for consecutive values of excitation for different stimuli. This effect arises because synchronizability of the network depends inherently on its connectivity structure, and the strength of synchrony for different stimuli is most diverse near and within the transition region. However, this also means that information entropy displays a sharp decline as network activity becomes strongly correlated for some stimuli, beginning within the transition region and reaching a minimum in the supercritical regions. Therefore, we propose that cortical networks operate at near-critical states, at the subcritical border of the transition region. Such marginally subcritical states allow for fine modulations of network excitability to dramatically enhance stimulus representation in the LFPs. In addition, for a putative encoding scheme in which higher area neurons integrate over the spiking activity in local V4 populations (coarse observation scale), near-critical states maximize information entropy.

### 3.1. Robustness of results

In this work we aimed to reproduce reproducing the characteristic features of the experimental findings with an uncomplicated model, in part due to considerations of computational expense. The conclusions of this paper depend mainly on the facts that in our model: (1) the emergence of synchronous spiking activity can be described by a phase transition as a function of an excitability parameter, and (2) synchronizability of the network depends implicitly on the topography of its connections. Therefore, we believe that as long as these requirements are met, discriminability enhancement will correlate with a narrow choice of parameters which generate near-critical dynamics. This will also be the case in more complex and biologically plausible models which detail different synchronization mechanisms which might be responsible for generating neural γ-activity (see, for example, the reviews Tiesinga and Sejnowski, 2009; Buzsáki and Wang, 2012).

In fact, recent modeling work by Poil et al. (2012), which employed a network consisting of IAF neurons with stochastic spiking and local connectivity, reported a result which nicely parallels our findings. For random realizations of their network architecture, the greatest variance of the power-law scaling of the avalanche size distributions was found near the critical points. In this framework, different random realizations of network connectivity were used to describe differences between human subjects, and the authors concluded that their findings provide an explanation for interindividual differences in α-oscillations in human MEG.

### 3.2. Physiological plausibility

We simulated cortical structure employing a random network of finite size, thus our model had a connectivity structure which varied for different subpopulations of activated neurons. This setting spared us any particular assumptions about the connection topology of V4 neurons, which is still subject of extensive anatomical research. In the brain, variability in connectivity of neurons in a local population is not random, but signifies a highly structured global network. Such functional connectivity is exemplified in the primary visual cortex by long-range connections between neurons with similar receptive field properties such as orientation preference (Kisvárday et al., 1997). These connections are thought to serve feature integration processes such as linking edge segments detected by orientation-selective neurons in V1 or V2 into more complex shapes, thus giving rise to the array of receptive field structures found in V4 (Desimone and Schein, 1987; David et al., 2006). In consequence, connection variability in the brain is significantly higher than random. Specifically, the variance of degree distributions is higher, the synaptic weights are heterogeneous, and the coupling structures are more anisotropic than in our simulations. Hence connection variability across different local networks is not decreased as drastically when the number of neurons is increased. In fact, assuming random variability implied a trade-off in our simulations: On the one hand, increasing the number of neurons decreased diversity in activation patterns and pattern separability, while on the other hand, it improved the assessment of criticality by increasing the range over which avalanche events could be observed.

In addition, in our model, we posited that attention modulates the efficacy of interactions, in order to reproduce the attention induced gain modulation and γ-synchrony using a reductionist approach. In biological networks, these effects may originate from more complicated mechanisms. For example, previous studies have shown that such an increase in gain (Chance et al., 2002) as well as synchronous activity (Buia and Tiesinga, 2006) can be achieved by modulating the driving background current. However, as described in Section 3.1, we expect our results will persist in other models where the network dynamics undergo a phase transition toward synchronous dynamics as a function of the responsiveness of neurons which is enhanced by attention. As an alternative to enhancing synaptic efficacy, we also tested a scenario in which attention provided an additional, weak external input to all neurons (results not shown). This led to qualitatively similar findings, with a quantitatively different discriminability boost.

Lastly, our current understanding of cortical signals strongly suggests that LFPs are generated mainly by a postsynaptic convolution of spikes from presynaptic neurons (Lindén et al., 2011; Makarova et al., 2014) and that even though other sources may contribute to the LFP signal, they are largely dominated by these synaptic transmembrane currents (Buzsáki et al., 2012). We generate the LFP signal through a convolution of the sum of appropriately scaled recurrent and external spiking activity. In our model, this closely approximates the sum of postsynaptic currents to V4 neurons: We are considering a very simple model of a small V4 population in which the postsynaptic potentials are evoked solely by these recurrent and external presynaptic spikes; degree distributions in the connectivity structure of the network has a small variance; the recurrent synaptic strengths are homogeneous; and there is no stochasticity in the recurrent synaptic transmission (i.e., every V4 spike elicits a postsynaptic potential in the V4 neurons it is recurrently coupled to). In addition, there is no heterogeneity in cell morphologies or the location of synapses, which are believed to influence the contribution of each synaptic current to the LFP signal in cortical tissue (Lindén et al., 2010). Combined, this means that each spike elicited by a model V4 neuron has a similar total impact on the postsynaptic membrane potentials, and the low-pass filtered spiking activity represents the postsynaptic currents well. Furthermore, even though our model does not incorporate the full biological complexity of cortical neurons, we believe that the particular choice of constructing the LFP signal in our model is not consequential for our results. The increase in discriminability of the LFP spectra originate primarily in the γ-band (both in the model and the experimental data), and we assume that correlated synaptic currents emerge simultaneously with correlated spiking activity, as there is experimental evidence that spiking (multi-unit) activity is synchronized with the LFP signal during attention-induced γ-oscillations (Fries et al., 2001).

### 3.3. Dynamics, structure, and function

In order to scrutinize the role of synchrony in enhancing stimulus representations, we considered an idealistic scenario: Each stimulus activates a different set with an *identical* number of neurons, so that without synchronization stimulus information encoded in activated neuron identities would be lost in the *average* population rate. By means of the different connectivities within different sets, however, this information becomes re-encoded in response amplitude and γ-synchrony. In principle, this concept is very similar to the old idea of realizing binding by synchrony (von der Malsburg, 1994), namely, using the temporal domain to represent information about relevant properties of a stimulus, for example, by tagging its features as belonging to the same object or to different objects in a scene.

However, strong synchronization hurts encoding by destroying information entropy. This is visible in the dynamics in the supercritical regime where ultimately all neurons do the same: fire together at identical times. Therefore, synchronization is only beneficial for information processing if additional constraints exist: for example, a neural bottleneck in which some aspect of the full information available would be lost, or a certain robustness of signal transmission against noise is required and can be realized by the synchronous arrival of action potentials at the dendritic tree.

In our setting, this bottleneck is the coarse observation scale where neuron identity information is lost by averaging over all neural signals. In such a case, information entropy is maximized as oscillations emerge at near-critical points. Although this situation is most dramatic for epidural LFPs that sum over thousands of neurons, it may also arise in more moderate scales if neurons in visual areas downstream of V4 have a large fan-in of their presynaptic connections. Naturally, this does not exclude the possibility that such a bottleneck may be absent and that cortical encoding can make use of spike patterns on finer spatial scales. This would shift the optimal operating regime “deeper” into the subcritical regime, and away from the transition region. Nonetheless, for this finer scale assumption, marginal subcriticality might represent a best-of-both-worlds approach. In particular, a penalty in information entropy may be necessary to ensure a certain level of synchronous activity required for other functionally relevant aspects of cortical dynamics, such as information routing regulated by attention via “communication through coherence” (Fries, 2005; Grothe et al., 2012).

In general, coding schemes being optimal for information transmission and processing always depends strongly on neural constraints and readout schemes. Nevertheless, specific assumptions about stimulus encoding do not influence our conclusion that the experimentally observed effects are unique to near-critical dynamics.

### 3.4. Outlook

In summary, our study establishes several, novel links between criticality, γ-synchronization, and task requirements (attention) in the mammalian visual system. Our model predicts that the cortical networks, specifically in visual area V4, operate at marginally subcritical regimes; task-dependent (e.g., attention induced) modulations of neuronal activity may push network dynamics toward a critical state; and the experimentally observed discriminability increase in LFP spectra can be attributed to differences in the network structure across different stimulus-specific populations. It remains for future studies to explore these links in more detail, and provide experimental support for our model's predictions. With recent advances in optogenetic methods and multielectrode recording techniques, assessing avalanche statistics in behaving, non-human primates with the required precision will soon be possible.

## 4. Materials and methods

### 4.1. Network model

#### 4.1.1. Structure and dynamics

The V4 network consists of *N* recurrently coupled IAF neurons *i* = 1, …, *N* described by their membrane potential *V*(*t*):

(5) $$\begin{array}{l} \tau_{mem}\frac{\text{d}V_{i}(t)}{\text{d}t}=-(V_{i}(t)-V_{R})+J_{ext}\sum_{k}\delta (t-{t^{\prime }}_{ik})\\ \;+J_{exc}\text{​}\sum_{j\;=\;1}^{N_{exc}}\text{​}w_{ij}\delta (t-t_{jk})-J_{inh}\text{​​​​}\sum_{j\;=\;N_{exc}\;+\;1}^{N}\text{​}\text{​​​}w_{ij}\delta (t-t_{jk}) \end{array}$$

The membrane potential evolves according to Equation 5 where every V4 neuron *i* has a resting potential *V*_*R*_ = −60 mV and generates an action potential when *V* crosses a threshold *V*_θ_ = −50 mV. After spiking, *V*(*t*) is reset back to *V*_*R*_. We picked the parameters to be representative of those of an average cortical neuron (Kandel et al., 2000; Noback et al., 2005). We used a membrane time constant of τ_*mem*_ = 10 ms. In Equation 5, *t*_*jk*_ denotes the *k*-th spike from V4 neuron *j*, and *t*′_*ik*_ the *k*-th spike from V1 (external input) to V4 neuron *i*.

V4 neurons are primarily driven by the external (feedforward) input once a stimulus is presented (see Section 4.1.2). Presynaptic V1 spikes have an external input strength *J*_*ext*_ = 0.1 mV.

*N*_*inh*_ V4 neurons are inhibitory (interneurons) and the remaining *N*_*exc*_ are excitatory cells (pyramidal neurons). We assumed a fixed ratio of ϵ = *N*_*exc*_/*N*_*inh*_ = 4 (Abeles, 1991). The neurons are connected via a random coupling matrix with connection probability *p* = 0.02 (Erdös-Renyi graph). Connections are directed (asymmetrical), and we allow for self-connectivity. *w*_*ij*_ assumes a value of 1 if a connection exists from neurons *j* to *i*, and is 0 otherwise. Global coupling strengths can independently be varied by changing *J*_*inh*_ and *J*_*exc*_.

Simulations were performed with an Euler integration scheme using a time step of Δ*t* = 0.1 ms. Membrane potentials of V4 neurons were initialized such that they would fire at random times (pulled from a uniform distribution) when isolated and driven by a constant input current (asynchronous state). We simulated the network's dynamics for a period of *T*_total_ = 2.5 s and discarded the first, transient 500 ms before analysis.

#### 4.1.2. Stimulus and external input

For comparison with the experimental data, we drove our network using *N*_*a*_ different stimuli. Specifically, we assumed that each stimulus activates a set of neurons in a lower visual area such as V1 or V2 whose receptive fields match (part of) the stimulus (Figure 1A). These neurons in turn provide feedforward input to a subset of *N*_active_ neurons in the V4 layer. We realized this input as independent homogeneous Poisson processes with rate *f*_*max*_ = 10 kHz. This situation is equivalent to each activated V4 neuron receiving feedforward input from roughly 1000 neurons, each firing at about 10 Hz during stimulus presentation.

Since stimuli used in the experiment had similar sizes, we assumed the subset of activated V4 neurons to have constant size *N*_*active*_ = 1000 for all stimuli. For each stimulus, we randomly choose the subset of V4 neurons which were activated by feedforward input. With a total of *N* = 2500 neurons, these subsets were not mutually exclusive for different stimuli. The remaining *N* − *N*_active_ neurons received no feedforward input. Each stimulus was presented to the network in *N*_*tr*_ independent trials, and the simulations were repeated for *N*_*w*_ independent realizations of the V4 architecture *w*_*ij*_.

#### 4.1.3. Local Field Potentials (LFPs)

In the experiments motivating this work, spiking activity was not directly observable. Only neural population activities (LFPs) were measured by epidural electrodes. Likewise, using our model we generated LFP signals *U*(*t*) by a linear superposition of spiking activities of all neurons *j* in layer V4 and spiking activities of V1 neurons presynaptic to V4 neurons *i*, scaled by a mixing constant of *c*_*mix*_ = 0.2. This was followed by a convolution with an exponential kernel *K*_*exp*_ (low-pass filter). In our network, this is a close approximation of summing the postsynaptic transmembrane currents of the V4 neurons (Lindén et al., 2011; Buzsáki et al., 2012; Makarova et al., 2014).

(6) $$U(t)=K_{exp}(t,\tau_{k})⊗\left(\text{​​}\sum_{jk}\delta (t-t_{jk})+c_{mix}\sum_{ik}\delta (t-{t^{\prime }}_{ik})\text{​​}\right)$$

(7) $$K_{exp}(t,\tau_{k})=\frac{1}{\tau_{k}}\;e^{-t/\tau_{k}}.$$

We used a time constant of τ_*k*_ = 15 ms for the kernel and discarded a period of 50 ms (~3.3 τ_*k*_) from both ends of the LFP signal in order to avoid boundary effects.

### 4.2. Analysis of network dynamics

#### 4.2.1. Spectral analysis

Mirroring the experiments, we performed a wavelet transform using complex Morlet's wavelets ψ(*t*, *f*) (Kronland-Martinet et al., 1987) for time-frequency analysis. We obtained the spectral power of the LFPs via

(8) $$p(t,f)={\left|\int_{-\infty }^{+\infty }\psi (\tau_{w},f)\;U(t-\tau_{w})\text{ d}\tau_{w}\right|}^{2}.$$

In order to exclude boundary effects, we only took wavelet coefficients outside the cone-of-influence (Torrence and Compo, 1998). Finally, we averaged the power *p*(*t*, *f*) over time to obtain the frequency spectra *p*(*f*). This method is identical to the one used for the analysis of the experimental data (Rotermund et al., 2009). The power *p*(*t*, *f*) of the signal was calculated in *N*_*f*_ = 20 different, logarithmically spaced frequencies *f*, in the range from *f*_*min*_ = 5 Hz to *f*_*max*_ = 200 Hz.

#### 4.2.2. Support vector machine classification

In order to assess the enhancement of stimulus representation in the LFPs, we performed SVM classification using the libsvm package (Chang and Lin, 2011). The SVM employed a linear kernel function and the quadratic programming method to find the separating hyperplanes. We implemented a leave-one-out routine, where we averaged over *N*_*tr*_ results obtained by using *N*_*tr*_ − 1 randomly selected trials for each stimulus for training and the remaining trial for testing.

#### 4.2.3. Discriminability index

The discriminability index DI(*J*_*exc*_, *j*_*inh*_) was defined as

(9) $$\begin{array}{l} \text{DI}=\frac{1}{N_{a}(N_{a}-1)/2}\frac{1}{N_{f}}\frac{1}{N_{tr}}\sum_{i\;=\;1}^{N_{a}-1}\sum_{j\;=\;i\;+\;1}^{N_{a}}\sum_{f}\sum_{tr}\\ \;\frac{\text{erf}(Z_{DI}(f,tr,i,j)/\sqrt{2})}{2}+\frac{1}{2} \end{array}$$

with

(10) $$Z_{DI}(f,tr,i,j)=\frac{\left|{\bar{p}}_{i}(f,tr)-{\bar{p}}_{j}(f,tr)\right|}{\sigma_{tr}({\bar{p}}_{i}(f,tr))+\sigma_{tr}({\bar{p}}_{j}(f,tr))}$$

where σ_*tr*_ is the standard deviation of frequency spectra *p* over different trials *tr* and erf(·) is the error function. The assumption underlying the DI measure is that, at a given frequency *f*, the magnitude of the LFP power distribution for different trials *tr* is normally distributed. Discriminability of two stimuli thus depend on how much the areas under their corresponding distributions overlap. DI represents the mean pairwise discriminability of unique stimulus pairs {*i*, *j*}, averaged over frequencies and trials. For one particular frequency band, the DI measure is related to the area-under-the-curve of a receiver-operator-characteristic of two normal distributions. By this definition, DI is normalized between 0.5 and 1, a higher DI indicating better discriminability. Because of trials having a finite duration, however, DI has a bias which took an approximate value of 0.69 in our simulations (Figures 3B, 4A, 6B). In addition, since there are typically frequencies which carry no stimulus information (e.g., the 110 Hz-band, see Figure 2B), DI is confined to values smaller than 1.

The discriminability index was further averaged over *N*_*w*_ independent realizations of the coupling matrix in the full simulations. In the reduced model, we ran the simulations for an extended duration of *T*_total_ = 12 s. For computing DI, we then divided the LFP time series into *N*_*tr*_ = 36 trials.

### 4.3. Neuronal avalanches

#### 4.3.1. Separation of time scales

A neuronal avalanche is defined as the consecutive propagation of activity from one unit to the next in a system of coupled neurons. The size of a neuronal avalanche is equal to the total number of neurons that are involved in that avalanche event, which starts when a neuron fires, propagates through generations of postsynaptic neurons, and ends when no neurons are activated anymore. Avalanche duration is then defined as the number of generations of neurons an avalanche event propagated through. In such a system, the critical point is characterized by a scale-free distribution of avalanche sizes and durations.

In simulations assessing avalanche statistics, recurrent spikes were delivered instantaneously to all postsynaptic neurons for proper separation of two different avalanches. This means that as soon as an avalanche event started, action potentials were propagated to all the generations of postsynaptic spikes within the same time step, until the avalanche event ended. This corresponds to a separation of timescales between delivery of external input and avalanche dynamics. In this way we could determine the avalanche sizes precisely, by “following” the propagation of every spike through the network.

In addition, we implemented a basic form of refractoriness which prevented a neuron from firing more than once during an avalanche event (holding its membrane potential at *V*_*R*_ after it fired). Since each avalanche event took place in a single time step of the simulations, this corresponded to each neuron having an effective refractory period equivalent to the integration time step Δ*t*.

#### 4.3.2. Analysis of criticality of dynamics

For each network realization, we obtained the probability *P*(*s*) of observing an avalanche of size *s* by normalizing histograms of avalanche sizes.

For every distribution *P*(*s*) obtained from our simulations, we calculated a maximum-likelihood estimator $\hat{\tau }$ for the power-law exponent τ using the statistical analysis described in Clauset et al. (2009) for discrete distributions. For a comprehensive account of the fitting method please see Clauset et al. (2009). To explain the procedure briefly, we started by defining a log-likelihood function 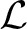
(τ). This quantifies the likelihood that the *n* empirical avalanche size observations *s*_*i*_ (*i* = 1, …, *n*), which were recorded during our simulations, were drawn from a perfect power-law distribution with exponent τ.

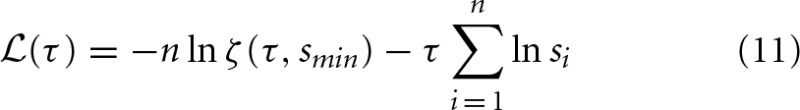



where

(12) $$\zeta (\tau ,s_{min})=\sum_{n\;=\;0}^{\infty }\;{\left(n+s_{min}\right)}^{-\tau }$$

is the Hurwitz zeta function. For a set of τ-values in the interval [1.1, 4], we computed 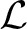
(τ) (using Equation 11) and the value of τ which maximized the log-likelihood was taken as the exponent $\hat{\tau }$ of the power-law fit *P*_*fit*_(*s*) ∝ *s*^−$\hat{\tau }$^. During the fitting procedure, we used a lower cut-off threshold *s*_*min*_ = *N*/100 = 10 and an upper cut-off threshold *s*_*max*_ = 0.6*N* = 600. In other words, we fit a power-law to the set of empirical observations in the interval *s*_*min*_ ≥ *s*_*i*_ ≥ *s*_*max*_. We repeated this fitting procedure to obtain power-law exponents α for the avalanche duration distributions *P*(*T*) ∝ *T*^−α^, using *T*_*min*_ = 5 and *T*_*max*_ = 30.

For clarity, it is important to point out that the ML analysis described in Clauset et al. (2009) does not take into consideration an upper cut-off in the empirical power-law distributions. One of the reasons we used an upper cut-off threshold during fitting is that the automated detection of critical points using the γ measure required us to fit a power-law exponent also to subcritical and supercritical avalanche size distributions. Using the complete tail of the distribution during the fitting procedure, for example in supercritical regimes, would yield a bias toward lower exponent estimates which would make it difficult to reliably detect the bump at large event sizes. This would hinder the detection of critical points using the γ measure, as it depends on an exponent which reliably represents the behavior of the distribution in the medium range of event sizes. More importantly, most of the size and duration distributions we observed at critical points displayed an exponential upper cut-off, as also observed in other experimental and theoretical work (Beggs and Plenz, 2003; Beggs, 2008; Petermann et al., 2009; Klaus et al., 2011; Arcangelis and Herrmann, 2012). In statistics of neuronal avalanches, the exact location of the cut-off threshold depends strongly on system size and the duration of observations, and increasing either will increase the number of sampled avalanches and shift the cut-off threshold to higher values, but not make it vanish. In addition, excluding the observations above a cut-off threshold reduced the absolute magnitude of the log-likelihood function for all values of τ (Equation 11), but the value of τ which maximized the log-likelihood provided us with a better estimate of the exponent for the middle range of the distributions where power-law scaling was prominent.

We used a least squares fitting procedure to find the power-law exponents for 〈*s*〉(*T*) (Weisstein, 2002), as it is not a probability distribution, using *T*_*min*_ = 2 and *T*_*max*_ = 20. In this procedure, the exponent 1/σν*z* of the function 〈*s*〉(*T*) ∝ *T*^1/σν*z*^ is given by the closed expression

(13) $$\frac{1}{\sigma \nu z}=\frac{m\sum_{i=1}^{m}\left(\ln T_{i}\ln{\langle s\rangle }_{i}\right)-\sum_{i=1}^{m}\left(\ln T_{i}\right)\sum_{i=1}^{m}\left(\ln{\langle s\rangle }_{i}\right)}{m\sum_{i=1}^{m}{\left(\ln T_{i}\right)}^{2}-\left(\sum_{i=1}^{m}{\left(\ln T_{i}\right)}^{2}\right)}\;\;\;$$

where *m* is the total number of points on the function 〈*s*〉(*T*), *T*_*i*_ are the duration values of the points and 〈*s*〉_*i*_ are the corresponding 〈*s*〉 values.

The KS statistic *D* was computed using

(14) $$D=\underset{{}_{s\;\geq \;N/100}}{\max}|F(s)-F_{fit}(s)|$$

where *F*(*s*) and *F*_*fit*_(*s*) are the cumulative distribution functions (CDFs) of *P*(*s*) and *P*_*fit*_(*s*), respectively.

We defined the transition region where the network dynamics switch from sub-critical to super-critical statistics using the binary variable indicator function γ.

(15) $$\gamma =\{\begin{array}{ccccc} 1 & \text{if} & F(N)-F(0.6N-1)>{F^{\prime }}_{fit}(N)-{F^{\prime }}_{fit}(0.6N-1) & & \text{​}\\ 0 & \text{ else} & \; & & \end{array}$$

In Equation 15, ${F^{\prime }}_{fit}(s)=F_{fit}(s)\;\frac{F(N/100)}{F_{fit}(N/100)}\;$. γ assumes a value of 1, signifying super-critical statistics, if the tail of the empirical avalanche size distributions *P*(*s* > 0.6*N*) is heavier than that of the fit. Additionally, we visually verified that the indicator γ works well for describing the behavior of the distributions in coupling space. The region in which its mean 〈γ〉 over *N*_*a*_ different stimuli lies between 0 and 1 was termed the transition region.

### 4.4. Computation of information entropy

We quantified information entropy *H*(**X**) using a state variable **X** which represents the spiking patterns of V4 neurons at a given time point *t* (Figure 1B). We construct the probability *P*(**X** = *x*_*i*_) of observing a spike pattern *x*_*i*_ using the *T*_total_Δ*t* spike patterns observed in one trial.

(16) $$H(X)=-\sum_{i}P(x_{i})\log_{2}P(x_{i})$$

Considering different read-out strategies of the information encoded by V4 neurons in the higher visual areas, we computed information entropy in different scales of observation *K*. These scales were defined as follows (Figure 1B):

For the finest observation scale, *K* = 1, the state variable **X** consists of *N* channels, representing *N* V4 neurons. Each channel assumes a value of 1 if the corresponding neuron generated an action potential at time *t*, and 0 otherwise. We randomly picked the order in which different neurons were represented in **X**.

As we increase the observation scale *K*, **X** comprises *N*/*K* channels, and each channel represents the sum of spikes from *K* different neurons. For *K* > 1, we constructed **X** by adding up the spiking activity of *K* consecutive neurons, while conserving the aforementioned random order of neurons over the channels. At the coarsest scale of observation, we sum over the activity of the whole network (i.e., for *K* = 1000, **X** is a scalar in the interval [0, 1000]).

## Funding

This work has been supported by the Bundesministerium für Bildung und Forschung (BMBF, Bernstein Award Udo Ernst, Grant No. 01GQ1106).

### Conflict of interest statement

The authors declare that the research was conducted in the absence of any commercial or financial relationships that could be construed as a potential conflict of interest.
